# Supplementary material for: Explainable machine learning model for predicting cesarean section following induction of labor: Development and external validation using real-world data
Source: PLOS Digit Health. 2025 Nov 20;4(11):e0001061. doi: 10.1371/journal.pdig.0001061 (PMC12633899; doi:10.1371/journal.pdig.0001061)
Supplement: S1 File — (DOCX) [file pdig.0001061.s001.docx]

**Explainable machine learning model for predicting cesarean section following induction of labor: Development and external validation using real-world data**

**Supplemental Material**

Table of Contents

[Section A 2](#_Toc210934328)

[Fig A: Inclusion and importance ranking of predictors of the seven models 3](#_Toc210934329)

[Fig B: Distribution of predicted probability in the temporal validation cohort 4](#_Toc210934330)

[Fig C: Composite maternal and neonatal morbidity by predicted probability decile group in the temporal validation cohort 5](#_Toc210934331)

[Fig D: Graphical abstract 6](#_Toc210934332)

[Fig E: Screenshots of the CSAI web application 7](#_Toc210934333)

[Table A: Data dictionary for candidate predictors 9](#_Toc210934334)

[Table B: Description of used machine learning algorithms 12](#_Toc210934335)

[Table C: Hyperparameter random search of used machine learning algorithms 13](#_Toc210934336)

[Table D: Glossary of terms used in clinical prediction modelling 14](#_Toc210934337)

[Table E: Sociodemographic and obstetric characteristics of included women 17](#_Toc210934338)

[Table F: Comparison of performance across seven models 19](#_Toc210934339)

[Table G: Comparison of method and performance between the XGBoost model and previously validated models 21](#_Toc210934340)

[References 23](#_Toc210934341)

# Section A

**Sample size calculation**

To assess whether our sample size of the training dataset was large enough to minimise model overfitting and to achieve sufficiently precise model predictions, we used the formula suggested in a recent sample size guideline for clinical prediction models.[^1^](#_ENREF_1) Based on the number of candidate predictors (n = 18) and proportion of outcome of interest (CS% = 20.8%), our sample size of 180,700 births was sufficient to target a mean absolute prediction error of 0.0025 between observed and true outcome probabilities. The number of births included in our validation cohorts also greatly exceeded the minimum sample size recommended for validation.[^2^](#_ENREF_2)

**Model training**

No data splitting (i.e. model training and testing samples) was performed for the development dataset, as recommended by the recent clinical prediction model guideline.[^1^](#_ENREF_1) Instead, each model was cross-validated, temporally and externally validated using separate datasets. No data balancing technique was applied as advised by the findings of prior comparison studies.[^3^](#_ENREF_3)^,^ [^4^](#_ENREF_4)

In the outer loop, hyperparameter tuning utilized ten random searches, each followed by five-fold cross-validation, with the area under the receiver operating characteristic curve (AUROC) as the scoring metric. Within each iteration of the outer loop, recursive feature elimination with cross-validation (RFECV) was performed on the selected four-fold training data, using an inner five-fold cross-validation (also scoring by AUROC), where one feature was removed at each time (step = 1), selecting the optimal subset of predictors for each hyperparameter configuration.

# Fig A: Inclusion and importance ranking of predictors of the seven models

**
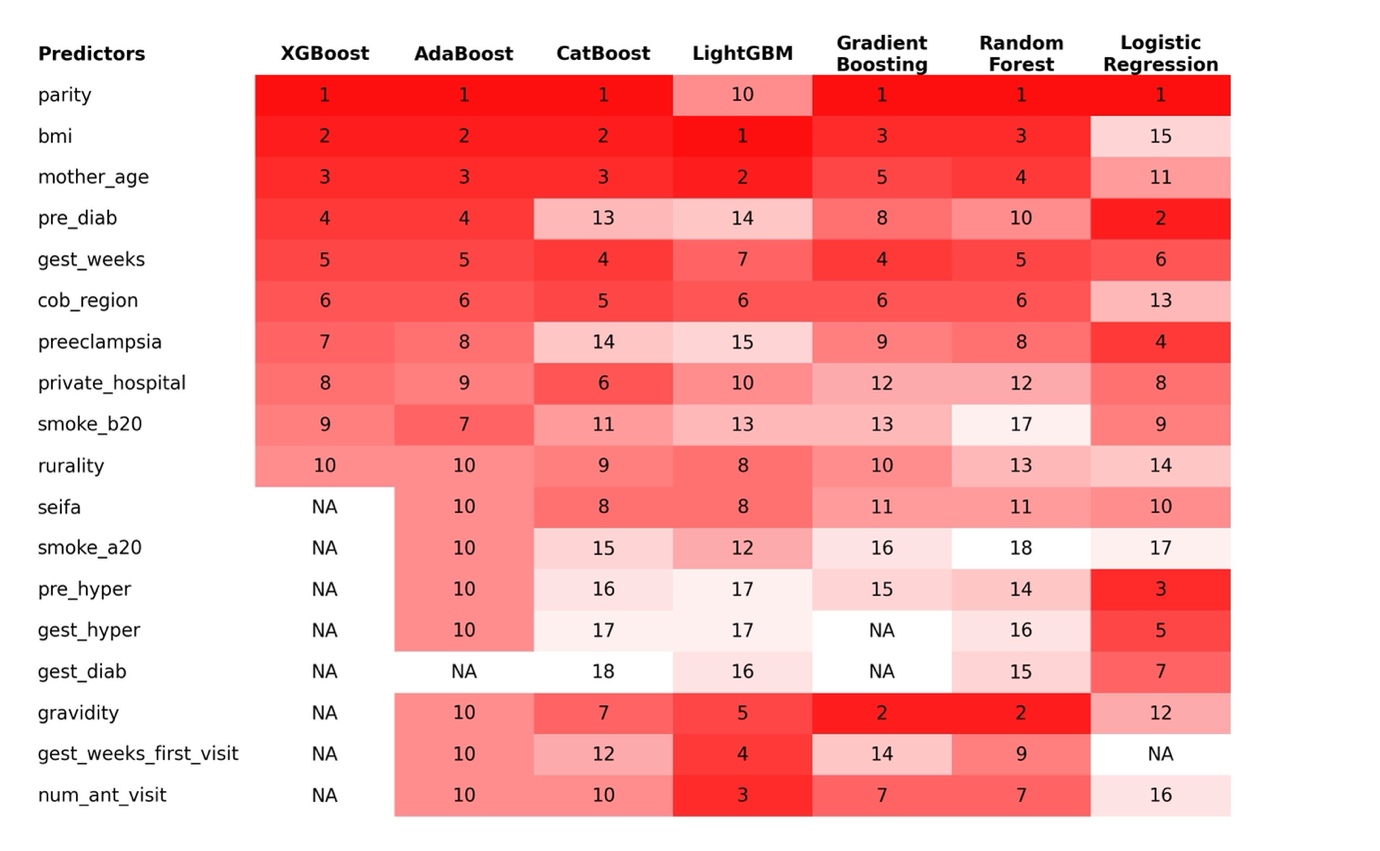
**

Predictors are ranked by *relative* importance specific to each model, with red indicating the highest importance and white indicating the lowest. NA (not applicable) means the model did not include this candidate predictor.

Definition and response options of each variable are detailed in Table A.

# Fig B: Distribution of predicted probability in the temporal validation cohort

**A. B.**


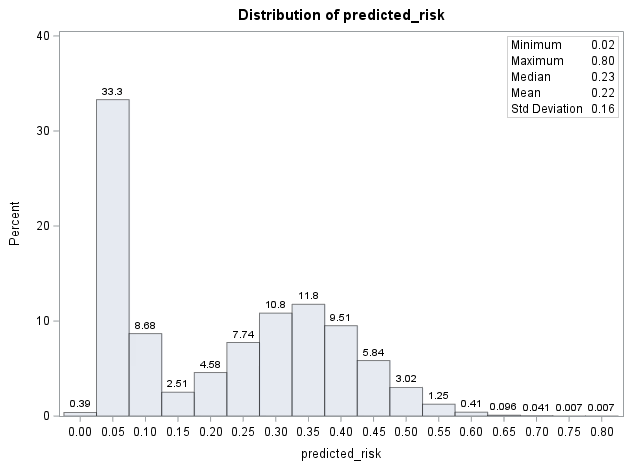

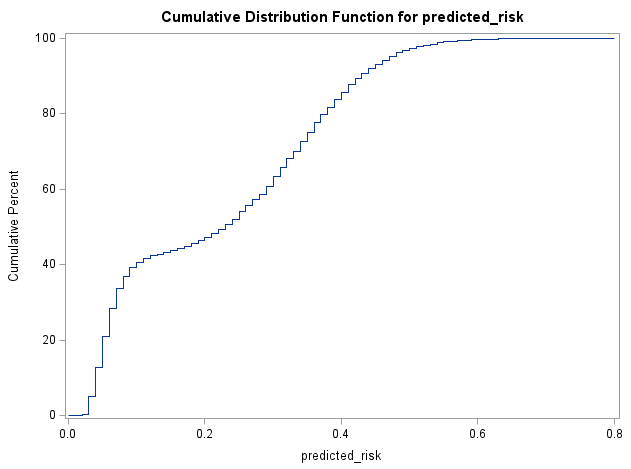


# Fig C: Composite maternal and neonatal morbidity by predicted probability decile group in the temporal validation cohort

**A. B.**


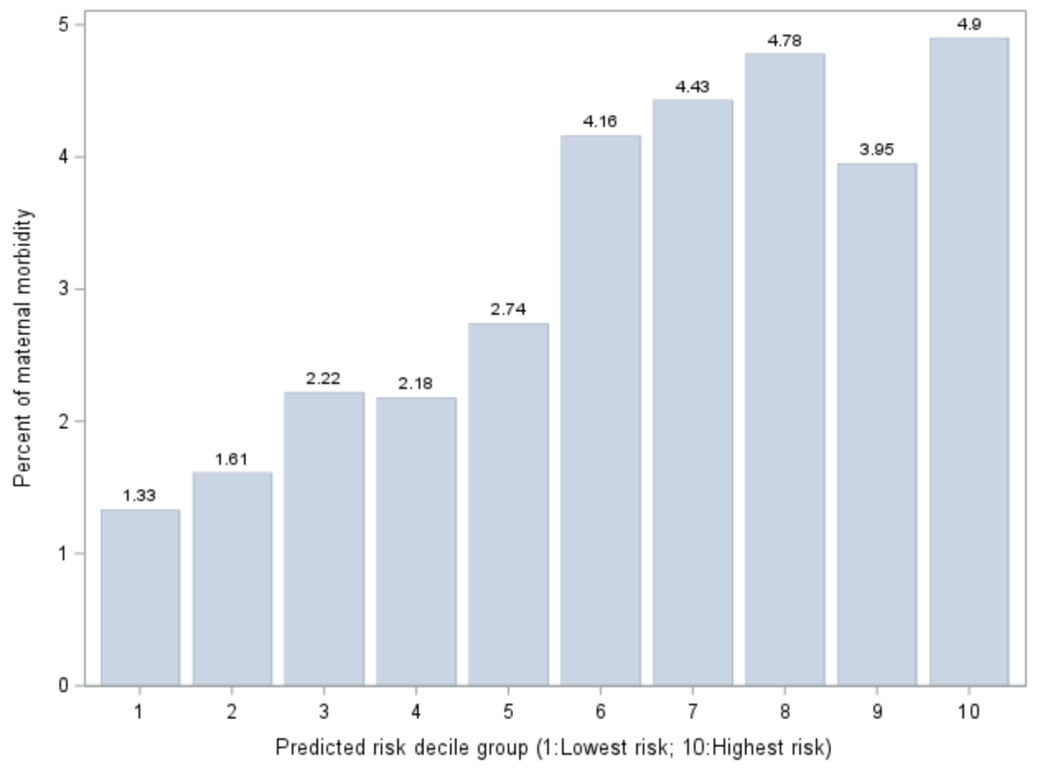

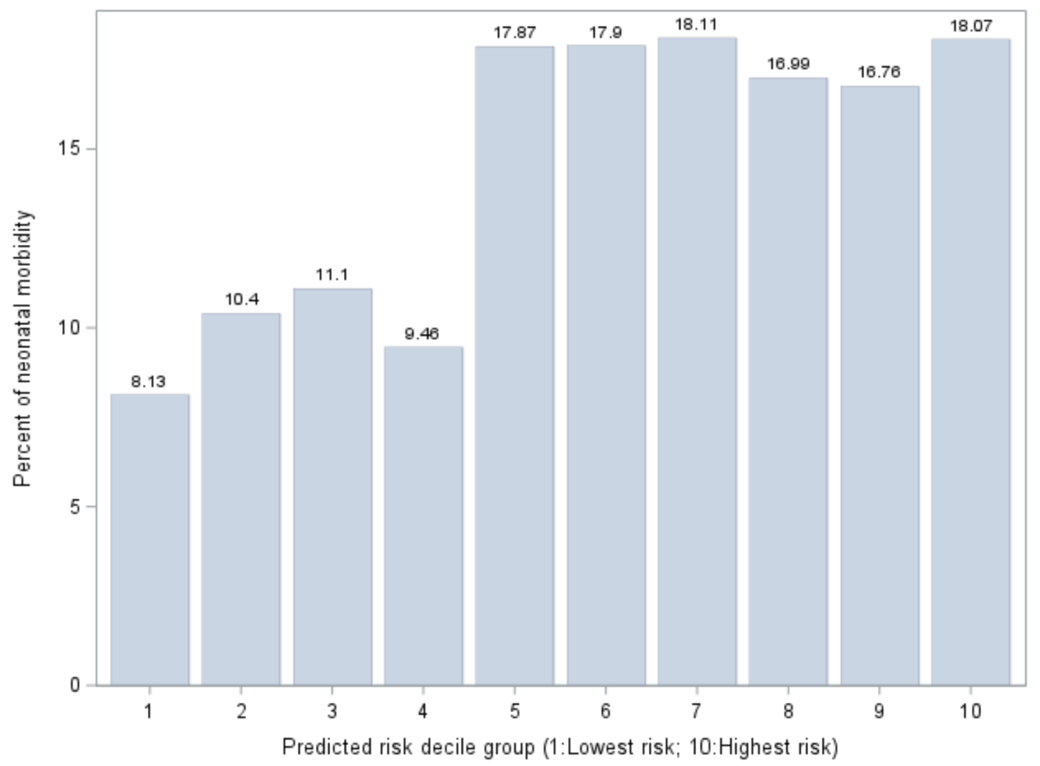


Composite maternal morbidity is defined by the occurrence of one or more of the following conditions from the time of giving birth to discharge: 3^rd^ or 4^th^ degree perineum tear, haemorrhage (ICD-10-AM code block: O67, O72), ruptured uterus (ICD-10-AM code block: O71), or retained placenta (ICD-10-AM code block: O73).

Composite neonatal morbidity is defined by the occurrence of one or more of the following conditions from birth to discharge: birth trauma––brachial plexus injury, fractured clavicle, or humorous (ICD-10-AM code block: P13.3, P13.4, P14), intrauterine hypoxia (ICD-10-AM code block: P20), hypoxic ischaemic encephalopathy (ICD-10-AM code block: P91.6), other perinatal morbidity––meconium aspiration syndrome, congenital pneumonia, or respiratory distress syndrome (ICD-10-AM code block: P24.0, P22, P23), APGAR Score < 7 at 5mins, or birthweight < 10^th^ percentile for their gestational age).

A higher predicted probability of caesarean section was associated with increased maternal and neonatal morbidity regardless of actual mode of birth.

# Fig D: Graphical abstract

# Fig E: Screenshots of the CSAI web application

**
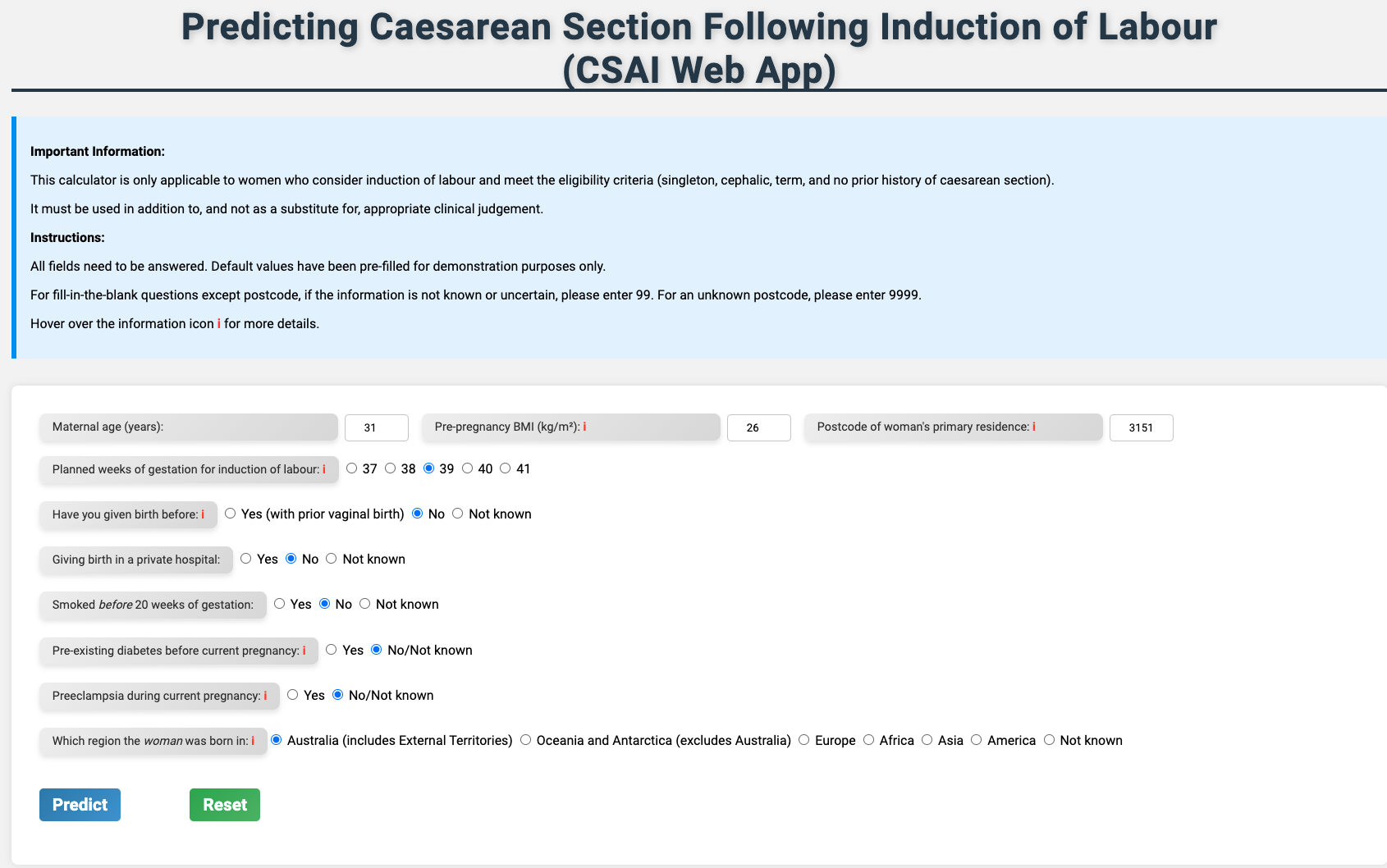
**

**
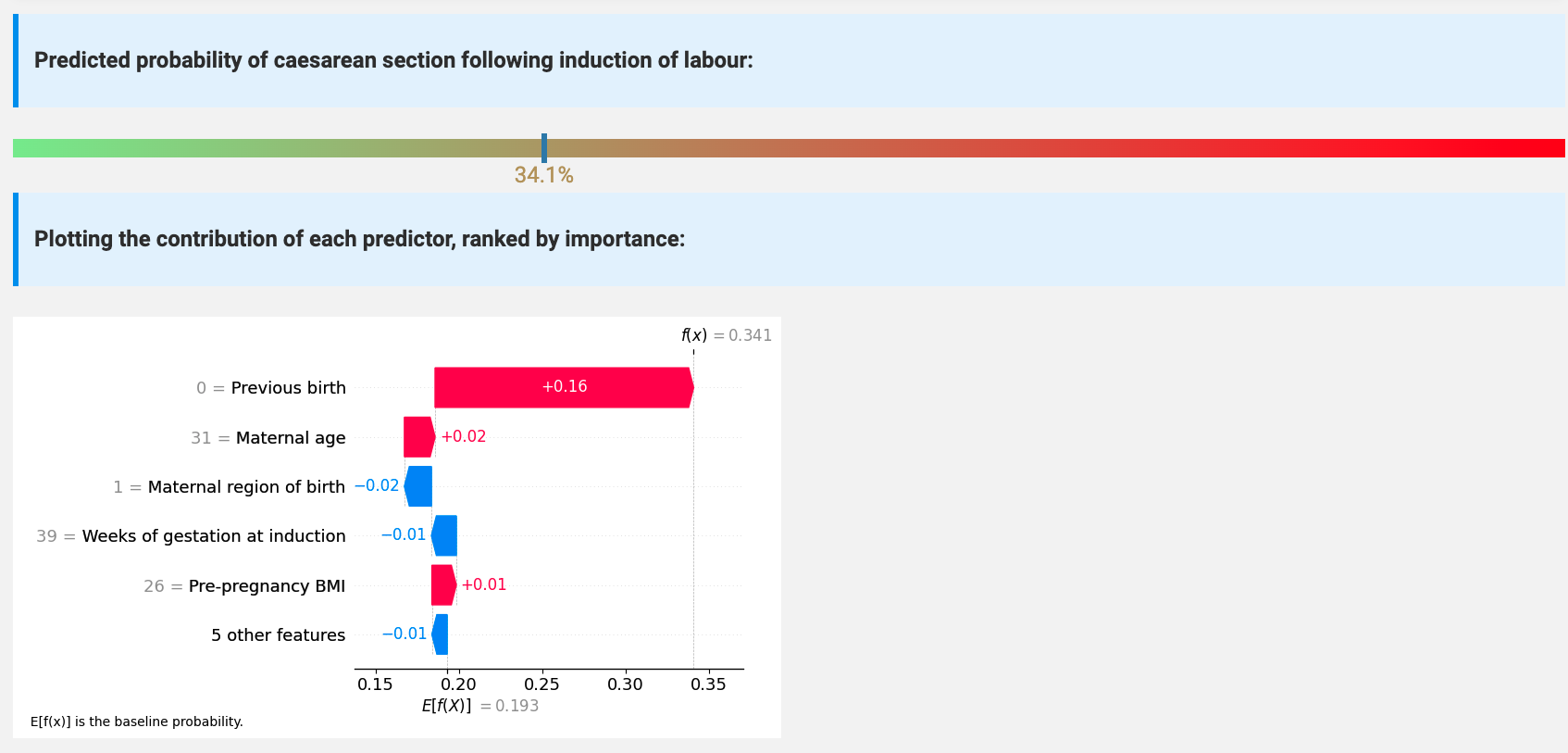
**

# Table A: Data dictionary for candidate predictors

| **No.** | **Variable name** | **Description** | **Format** | **Response options** | **Note** |
| --- | --- | --- | --- | --- | --- |
|  | bmi | Women’s BMI (kg/m^2^) | Continuous | 99 = Not stated/missing | Weights were collected at different time points in NPDC (record in the first trimester of pregnancy), VPDC (self-reported at conception) and QPDC (self-reported four to six weeks prior to or at conception). |
|  | cob_region | Women’s country of birth grouped into broad regions | Categorical | 1 = Australia (includes External Territories)  2 = Oceania and antarctica (excludes Australia)  3 = Europe  4 = Africa  5 = Asia  6 = America  9 = Not stated/missing | Women’s country of birth was categorized based on the Standard Australian Classification of Countries (SACC) used by the Australian Bureau of Statistics (ABS).[^5^](#_ENREF_5) |
|  | gest_diab | Gestational diabetes during current pregnancy | Dichotomous | 1 = Yes  0 = No/ not stated/missing | As collected and defined in the NPDC and VPDC.  O244 (ICD-10-AM code) reported in pregnancy complications collected in the QPDC. |
|  | gest_hyper | Gestational hypertension during current pregnancy (excludes preeclampsia) | Dichotomous | 1 = Yes  0 = No/ not stated/missing | As collected and defined in the NPDC and VPDC.  O13 (ICD-10-AM code) reported in pregnancy complications in the QPDC. |
|  | gest_weeks | Weeks of gestation at birth | Categorical | 37 = 37^+0^–37 ^+6^  38 = 38^+0^–38^+6^  39 = 39^+0^–39^+6^  40 = 40^+0^–40^+6^  41 = 41^+0^–41^+6^  99 = Not stated/missing | As collected and defined in the NPDC, VPDC and QPDC. |
|  | gest_weeks_first_visit | Weeks of gestation at first antenatal visit | Continuous | 99 = Not stated/missing | As collected and defined in the NPDC, VPDC and QPDC. |
|  | gravidity | Number of previous pregnancies | Continuous | 99 = Not stated/missing | As collected and defined in the NPDC, VPDC and QPDC. |
|  | mother_age | Women’ age at time of giving birth (years) | Continuous | 99 = Not stated/missing | As collected and defined in the NPDC, VPDC and QPDC. |
|  | num_ant_visit | Number of total antenatal visits | Continuous | 99 = Not stated/missing | As collected and defined in the NPDC, VPDC and QPDC. |
|  | parity | Whether women gave birth before current pregnancy (live births or stillbirths of at least 20 weeks of gestation and/or at least 400 grams in weight) | Categorical | 0 = No (nulliparous)  1 = Yes (parous with prior vaginal birth)  9 = Not stated/missing | As collected and defined in the NPDC, VPDC and QPDC. |
|  | pre_diab | Pre-existing diabetes (any type) | Dichotomous | 1 = Yes  0 = No/ not stated/missing | As collected and defined in the NPDC and VPDC.  E10-E14 or O24 (ICD-10-AM code) reported in current medical conditions in the QPDC. |
|  | pre_hyper | Pre-existing hypertension (any type) | Dichotomous | 1 = Yes  0 = No/ not stated/missing | As collected and defined in the NPDC and VPDC.  I11-I15 or O10 or O11 (ICD-10-AM code) reported in current medical conditions in the QPDC. |
|  | preeclampsia | Preeclampsia during current pregnancy | Dichotomous | 1 = Yes  0 = No/ not stated/missing | As collected and defined in the NPDC and VPDC.  O11, O14 or O15 (ICD-10-AM code) reported in current medical conditions in the QPDC. |
|  | private_hospital | Giving birth in private or public hospitals | Categorical | 1 = Yes  0 = No  9 = Not stated/missing | As collected and defined in the NPDC, VPDC and QPDC. |
|  | rurality | Women’s residential area’s rurality group | Ordinal | 1 = Major cities of Australia  2 = Inner regional Australia  3 = Outer regional Australia  4 = Remote Australia  5 = Very remote Australia  9 = Not stated/missing/migratory-offshore-shipping/no usual address | Rurality was categorized based on the postcode of usual residence using the Accessibility/Remoteness Index of Australia (ARIA+) used by the Australian Bureau of Statistics (ABS).[^6^](#_ENREF_6)  2011 release was used by the NPDC. 2016 release was used for VPDC and QPDC. |
|  | seifa | Women’s residential area of socioeconomic disadvantage | Ordinal | 1 = Quintile 1 (the most disadvantaged)  2 = Quintile 2  3 = Quintile 3  4 = Quintile 4  5 = Quintile 5 (the least disadvantaged)  9 = Not stated/missing | Socioeconomic status was categorized based on the postcode of usual residence using the Index of Relative Socio-economic Disadvantage (IRSD) in the Socio-Economic Indexes for Areas (SEIFA)–a classification system developed by the Australian Bureau of Statistics (ABS) that ranks geographic areas across Australia according to relative socio-economic advantage and disadvantage.[^7^](#_ENREF_7)  2011 release was used by the NPDC. 2016 release was used for VPDC and QPDC. |
|  | smoke_a20 | Smoke after 20 weeks of gestation | Categorical | 1 = Yes  0 = No  9 = Not stated/missing | As collected and defined in the NPDC, VPDC and QPDC. |
|  | smoke_b20 | Smoke before 20 weeks of gestation | Categorical | 1 = Yes  0 = No  9 = Not stated/missing | As collected and defined in the NPDC, VPDC and QPDC. |

Abbreviations: BMI: Body Mass Index; NPDC: New South Wales Perinatal Data Collection; VPDC: Victoria Perinatal Data Collection; QPDC: Queensland Perinatal Data Collection.

Perinatal data collection manuals for each state are available at <https://github.com/Yanan-Hu/CSAI/tree/main/Data%20dictionary>.

# Table B: Description of used machine learning algorithms

| **Algorithm** | **Description** |
| --- | --- |
| Logistic Regression | A linear model that uses the logit function to model the relationship between predictors and the outcome. Due to its simplicity and interpretability, it is often used as a baseline model, suitable for scenarios where the relationship between predictors and the outcome is approximately linear but may not capture complex interactions. |
| Random Forest Classifier | A parallel ensemble learning method that constructs multiple independent decision trees using bootstrapped samples of the training data and random subsets of predictors. Each tree votes on the outcome, and the final classification is determined by majority vote. It is robust against overfitting, can handle complex interactions between predictors, and work well on high-dimensional datasets. However, training multiple trees increases computation time and memory usage. |
| Gradient Boosting Classifier | A sequential ensemble method that builds an ensemble of weak decision trees, where each new tree corrects the residual errors of the previous ones by optimizing a loss function. It is particularly effective for structured/tabular data and scenarios with complex interactions between predictors. |
| LightGBM (Light Gradient-Boosting Machine) Classifier | A variant of gradient boosting that uses histogram-based binning and a leaf-wise tree growth strategy to improve computational efficiency and memory usage. It is well-suited for large datasets with many predictors, as it can handle high-dimensional data effectively without sacrificing accuracy. |
| CatBoost (Categorical Boosting) Classifier | A variant of gradient boosting that handles categorical predictors natively using an innovative ordered boosting process to mitigate overfitting and improve generalization. It is particularly powerful for datasets with many categorical variables and missing values, as it requires minimal preprocessing like one-hot encoding or imputation, but can be slower to train compared to other boosting methods. |
| XGBoost (eXtreme Gradient Boosting) Classifier | A variant of gradient boosting that incorporates advanced split-finding algorithms, sparsity-aware optimization, and regularization techniques to prevent overfitting. It is highly effective for structured/tabular data with complex interactions between predictors, offering a good balance between speed and performance. |
| AdaBoost (Adaptive Boosting) Classifier | A sequential ensemble method that trains a series of weak learners (shallow decision trees), focusing on the hardest-to-classify samples by adjusting their weights. The final model combines the weighted predictions of all weak learners to produce a strong classifier that minimizes classification error. It is effective for improving the performance of simple classifiers on small to moderate-sized datasets but is sensitive to noisy data and outliers. |

# Table C: Hyperparameter random search of used machine learning algorithms

| **Classification algorithm** | **Hyperparameter random search** | **Selected hyperparameter** |
| --- | --- | --- |
| Logistic Regression | C: uniform (0.01, 10)  penalty: ['l1', 'l2', 'elasticnet', None]  solver: ['sag', 'saga', 'liblinear', 'lbfgs', 'newton-cg'] | C: 9.40  penalty: l2  solver: lbfgs |
| Random Forest | n_estimators: randint (50, 150)  max_depth: randint (3, 10)  min_samples_leaf: randint (20, 100)  min_samples_split: randint (20, 100)  max_features: ['sqrt','log2'] | n_estimators: 104  max_depth: 8  min_samples_leaf: 81  min_samples_split: 70  max_features: sqrt |
| Gradient Boosting | n_estimators: randint (50, 150)  learning_rate: uniform (0.01, 0.5)  max_depth: randint (3, 10)  min_samples_leaf: randint (20, 100)  min_samples_split: randint (20, 100)  subsample: uniform (0.5, 0.5)  max_features: ['sqrt','log2'] | n_estimators: 70  learning_rate: 0.27  max_depth: 3  min_samples_leaf: 70  min_samples_split: 26  subsample: 0.73  max_features: sqrt |
| LightGBM | n_estimators: randint (50, 150)  learning_rate: uniform (0.01, 0.5)  num_leaves: randint (20, 100)  max_depth: randint (3, 10)  min_child_samples: randint (20, 100)  subsample: uniform (0.5, 0.5)  colsample_bytree: uniform (0.5, 0.5) | n_estimators: 100  learning_rate: 0.50  num_leaves: 26  max_depth: 3  min_child_samples: 22  subsample: 0.59  colsample_bytree: 0.69 |
| CatBoost | iterations: randint (50, 150)  learning_rate: uniform (0.01, 0.5)  depth: randint (3, 10)  l2_leaf_reg: uniform (1, 10)  bagging_temperature: uniform (0, 1) | iterations: 104  learning_rate: 0.24  depth: 5  l2_leaf_reg: 10.83  bagging_temperature: 0.79 |
| XGBoost | n_estimators: randint (50, 150)  learning_rate: uniform (0.01, 0.5)  max_depth: randint (3, 10)  subsample: uniform (0.5, 0.5)  colsample_bytree: uniform (0.5, 0.5)  gamma: uniform (0, 5) | n_estimators: 52  learning_rate: 0.50  max_depth: 3  subsample: 0.93  colsample_bytree: 0.81  gamma: 1.91 |
| AdaBoost | n_estimators: randint (50, 150)  learning_rate: uniform (0.01, 0.5)  algorithm: ['SAMME','SAMMME.R'] | n_estimators: 79  learning_rate: 0.49  algorithm: SAMME |

All others used the default value.

# Table D: Glossary of terms used in clinical prediction modelling

| **Term** | **Explanation** |
| --- | --- |
| Outcome (also known as class label, target variable, or dependent variable) | The predicted probability of whether a woman will undergo a primary caesarean section following induction of labour. |
| Predictors (also known as covariates, independent variable, baseline variables, or features) | A set of woman-level characteristic, including demographic, medical, and obstetric characteristics, that may influence the outcome. |
| Candidate predictors (also known as potential predictors) | Variables that may potentially be used to predict the outcome. |
| Included predictors (also known as selected predictors) | The specific subset of candidate predictors that are included in the prediction model based on their relevance to the outcome. |
| Data balancing (also known as class balancing) | Techniques used to adjust the training dataset to ensure an equal representation of different outcome classes. |
| Training dataset (also known as development dataset) | The data used to develop the prediction model. |
| Hyperparameter | Configuration settings that are set before the learning process begins and govern the training process (e.g. learning rate or maximum depth of a tree). |
| Hyperparameter tuning (also known as hyperparameter optimization) | The process of optimizing the settings of the algorithms used in the model to improve prediction performance. |
| Randomised hyperparameter search | A method of hyperparameter tuning that randomly samples combinations of hyperparameters to identify the best-performing model configuration. |
| Feature selection | The method of selecting a subset of candidate predictors for inclusion in the model, ensuring that only the most impactful variables are used to predict the outcome. |
| Data processing pipeline | A sequence of data processing steps, including hyperparameter tuning, feature selection, model training, and evaluation, designed to streamline the prediction process. |
| Parameters (also known as coefficients or weights) | Values that are adjusted by the learning algorithm during training to minimize the loss function (e.g. the coefficients in logistic regression). |
| Relative importance | A measure of how important each predictor is in the model's predictions. Different models may calculate relative importance using various metrics, such as the change in model accuracy or loss when a predictor is permuted or removed. |
| Internal validation | Methods used to assess the performance of prediction models using the data from which the model was developed, providing an honest estimate of predictive accuracy. |
| Split sample internal validation (also known as holdout validation or testing) | An internal validation method where the dataset is randomly divided into two parts; one part is used for model development, while the other assesses model performance. |
| k-fold cross-validation | The dataset is randomly divided into k folds; the model is trained on k−1 folds and tested on the remaining fold, cycling through all folds to validate model performance. |
| Temporal validation (also known as time-based validation) | A validation approach where data is divided based on the timing of events; the model is trained on women who gave birth earlier and validated on those who gave birth later. |
| Geographical validation (also known as spatial validation) | Externally validating the model's predictions using data from different geographical settings. |
| Bootstrapping (also known as resampling) | A resampling technique that creates multiple samples from the original dataset by drawing with replacement, allowing for generating the confidence interval (uncertainty) of the model's performance. |
| Underfitting | When a model is too simple to capture the underlying patterns in the data effectively, leading to poor performance across both the training and validation datasets. |
| Overfitting | When a model learns the training data too well, capturing noise and random fluctuations rather than true patterns, especially when the sample size is small relative to the number of parameters. This results in high performance on the training dataset but poor predictive performance on new, unseen data, creating optimism based on the training results. |
| Optimism | Difference between a model's performance on the training dataset and on new data. |
| Model interpretability (also known as model explainability) | The degree to which the predictions of the model can be understood and explained, critical for clinical acceptance and trust. |
| SHAP (SHapley Additive exPlanations) | A method to explain individual predictions by calculating the contribution of each predictor based on cooperative game theory. Specifically, SHAP values use the concept of Shapley values, which assign a fair distribution of the total payoff (the predicted probability) among all players (predictors) based on their contributions. |
| Model performance | Evaluation metrics used to assess how well the model predicts the outcome, including accuracy, precision, recall, and others. |
| Discrimination | The model's ability to assign higher predicted probabilities to women who had a caesarean section than those who did not. |
| Calibration | The agreement between predicted probabilities and observed outcomes. A calibration curve can be created to visually assess this relationship by grouping participants based on predicted probabilities. |
| Brier score | A metric measuring overall performance, calculated as the mean squared difference between predicted probabilities and actual outcomes. It provides a unified measure of discrimination and calibration. |
| Decision curve analysis | A method to evaluate the clinical utility of the prediction model by considering the net benefits of different threshold probabilities for decision-making. The treatment or intervention could be any action that a woman with a high predicted risk would consider to improve her health. |
| Treat all (also known as universal treatment) | A strategy where a*ll* women receive the intervention (e.g. closer monitoring or alternative treatment), regardless of model predictions. |
| Treat none (also known as no treatment) | A strategy where *no* women receive the intervention (e.g. closer monitoring or alternative treatment), regardless of model predictions. |
| Threshold probability (also known as cut-off probability) | The specific predicted probability level at which the decision-maker would take a given action. Selecting a decision threshold can be subjective, and the appropriate range of thresholds may vary based on factors such as clinical settings, available treatment options, and the preferences of women. |
| Net benefit | The net benefit is computed as the expected proportion of true positives (identified and appropriately managed) minus the expected proportion of false positives (unnecessary action), adjusted by a weight based on the selected cut-off threshold. This metric is useful for evaluating the clinical value of the prediction model by measuring the trade-off between true positive outcomes and the harms of false positives. |
| Reproducibility | The ability to consistently replicate the estimated model performance in new samples from the same population, reinforcing the reliability of predictions. |
| Transportability | The capability of the model to provide accurate predictions for new women drawn from a different but related population or setting, ensuring relevance across diverse clinical contexts. |
| Generalisability | The overall ability of the model to be applied beyond the original training dataset, encompassing both reproducibility and transportability. |
| Web application | A software application that enables users to input data and receive predictions from the model, facilitating practical use of the prediction tool in clinical settings. |

# Table E: Sociodemographic and obstetric characteristics of included women

| **Characteristics**  N, % | **Training cohort** | **Temporal validation cohort** | **Geographical validation cohort**^a^ |
| --- | --- | --- | --- |
| **All** | **180,700** | **14,527** | **14,755** |
| **Women’s age at time of giving birth** (years) | | | |
| ≤ 19 | 3,542 (1.96) | 155 (1.07) | 127 (0.86) |
| 20–34 | 132,403 (73.27) | 10,215 (70.32) | 10,335 (70.04) |
| ≥ 35 | 44,734 (24.76) | 4,154 (28.6) | 4,293 (29.1) |
| Missing | 21 (0.01) | 3 (0.02) | 0 |
| Mean ± Standard deviation | 30.69 ± 5.48 | 31.45 ± 5.33 | 31.81 ± 4.9 |
| **Pre-pregnancy BMI** (kg/m^2^) | | | |
| < 18.5 (Underweight) | 7,009 (3.88) | 482 (3.32) | 639 (4.33) |
| 18.5–24.9 (Normal weight) | 85,033 (47.06) | 6,872 (47.31) | 8,172 (55.38) |
| 25.0–29.9 (Overweight) | 43,855 (24.27) | 3,626 (24.96) | 3,811 (25.83) |
| ≥ 30.0 (Obesity) | 41,198 (22.8) | 3,222 (22.18) | 2,059 (13.95) |
| Missing | 3,605 (2) | 325 (2.24) | 74 (0.5) |
| Mean ± Standard deviation | 26.31 ± 6.4 | 26.34 ± 6.33 | 24.76 ± 5.42 |
| **Women’s country of birth** (grouped in regions) | | | |
| Australia (Includes External Territories) | 121,814 (67.41) | 9,232 (63.55) | 9,826 (66.59) |
| Oceania and Antarctica (Excludes Australia) | 7,868 (4.35) | 404 (2.78) | 342 (2.32) |
| Europe | 8,782 (4.86) | 734 (5.05) | 465 (3.15) |
| Africa | 3,601 (1.99) | 289 (1.99) | 381 (2.58) |
| Asia | 34,841 (19.28) | 3,528 (24.29) | 2,317 (15.7) |
| America | 3,286 (1.82) | 298 (2.05) | 216 (1.46) |
| Missing | 508 (0.28) | 42 (0.29) | 1,208 (8.19) |
| **Women’s residential area of socioeconomic disadvantage (**SEIFA) | | | |
| 1^st^ quintile (the most disadvantaged) | 35,627 (19.72) | 3,092 (21.28) | 1,393 (9.44) |
| 2^nd^ quintile | 38,094 (21.08) | 3,591 (24.72) | 2,332 (15.8) |
| 3^rd^ quintile | 39,636 (21.93) | 2,883 (19.85) | 3,022 (20.48) |
| 4^th^ quintile | 35,595 (19.7) | 2,484 (17.1) | 3,595 (24.36) |
| 5^th^ quintile (the least disadvantaged) | 29,966 (16.58) | 2,314 (15.93) | 4,413 (29.91) |
| Missing | 1,782 (0.99) | 163 (1.12) | 0 |
| **Women’s residential area of rurality** | | | |
| Major cities of Australia | 131,330 (72.68) | 11,261 (77.52) | 11,581 (78.49) |
| Inner regional Australia | 28,272 (15.65) | 2,293 (15.78) | 2,504 (16.97) |
| Outer regional Australia | 13,869 (7.68) | 617 (4.25) | 656 (4.45) |
| Remote Australia | 1,612 (0.89) | 50 (0.34) | 5 (0.03) |
| Very remote Australia | 915 (0.51) | 15 (0.1) | 9 (0.06) |
| Missing | 4,702 (2.6) | 291 (2) | 0 |
| **Smoking status before 20 weeks of gestation** | | | |
| No | 163,915 (90.71) | 13,289 (91.48) | 13,758 (93.24) |
| Yes | 16,429 (9.09) | 1,200 (8.26) | 740 (5.02) |
| Missing | 356 (0.2) | 38 (0.26) | 257 (1.74) |
| **Smoking status after 20 weeks of gestation** | | | |
| No | 166,809 (92.31) | 13,490 (92.86) | 13,315 (90.24) |
| Yes | 12,779 (7.07) | 934 (6.43) | 399 (2.7) |
| Missing | 1,112 (0.62) | 103 (0.71) | 1,041 (7.06) |
| **Birthplace of child** | | | |
| Public hospital | 130,554 (72.25) | 11,401 (78.48) | 8,265 (56.01) |
| Private hospital | 49,884 (27.61) | 3,126 (21.52) | 6,431 (43.59) |
| Missing | 262 (0.14) | 0 | 59 (0.4) |
| **Weeks’ gestation at birth** | | | |
| 37 | 17,216 (9.53) | 1,185 (8.16) | 1,022 (6.93) |
| 38 | 40,718 (22.53) | 2,955 (20.34) | 2,909 (19.72) |
| 39 | 50,974 (28.21) | 4,746 (32.67) | 3,766 (25.52) |
| 40 | 40,829 (22.59) | 3,372 (23.21) | 3,812 (25.84) |
| 41 | 30,963 (17.14) | 2,269 (15.62) | 3,246 (22) |
| **Weeks of gestation at first antenatal visit** | | | |
| Missing | 414 (0.23) | 61 (0.42) | 90 (0.61) |
| Mean ± Standard deviation | 10.27 ± 5.65 | 9.38 ± 5.46 | 11.72 ± 5.98 |
| **Number of total antenatal visits** | | | |
| Missing | 313 (0.17) | 31 (0.21) | 234 (1.59) |
| Mean ± Standard deviation | 10.92 ± 3.84 | 10.54 ± 3.93 | 9.58 ± 3.41 |
| **Number of previous pregnancies** | | | |
| Missing | 62 (0.03) | 0 | 0 |
| Mean ± Standard deviation | 1.04 ± 1.48 | 0.76 ± 1.13 | 1.16 ± 1.37 |
| **Parity** | | | |
| Nulliparous | 96,843 (53.59) | 8,140 (56.03) | 8,618 (58.41) |
| Parous with prior vaginal birth | 83,807 (46.38) | 6,387 (43.97) | 6,137 (41.59) |
| Missing | 50 (0.03) | 0 | 0 |
| **Pre-existing diabetes (any type)** | | | |
| No/missing | 178,911 (99.01) | 14,384 (99.02) | 14,755 (100) |
| Yes | 1,789 (0.99) | 143 (0.98) | 0 |
| **Pre-existing hypertension (any type)** | | | |
| No/missing | 178,724 (98.91) | 14,337 (98.69) | 14,755 (100) |
| Yes | 1,976 (1.09) | 190 (1.31) | 0 |
| **Gestational diabetes** | | | |
| No/missing | 147,479 (81.62) | 11,855 (81.61) | 14,755 (100) |
| Yes | 33,221 (18.38) | 2,672 (18.39) | 0 |
| **Gestational hypertension** (excludes preeclampsia) | | | |
| No/missing | 170,763 (94.4.5) | 13,945 (95.99) | 14,755 (100) |
| Yes | 9,937 (5.5) | 582 (4.4.01) | 0 |
| **Preeclampsia** | | | |
| No/missing | 175,659 (97.21) | 14,196 (97.72) | 14,755 (100) |
| Yes | 5,041 (2.79) | 331 (2.28) | 0 |
| **Mode of birth** | | | |
| Vaginal birth | 143,176 (79.23) | 11,259 (77.5) | 11,959 (81.05) |
| Caesarean section | 37,524 (20.77) | 3,268 (22.5) | 2,796 (18.95) |

^a^For the geographical validation cohort, we were only able to identify women who did not have any of these conditions owing to the availability of our dataset. The mean and standard deviation were calculated for births with non-missing data only.

Abbreviations: BMI: Body Mass Index; SEIFA: Socio-Economic Indexes for Areas.

# Table F: Comparison of performance across seven models

| **Performance metric** | | **Classification algorithm** | | | | | | | **Rank of XGBoost** |
| --- | --- | --- | --- | --- | --- | --- | --- | --- | --- |
|  |  | **Logistic Regression** | **Random Forest** | **Gradient Boosting** | **LightGBM** | **CatBoost** | **XGBoost** | **AdaBoost** |  |
| Overall training time (hours)^a^ $\downarrow$ | | 4 | 23 | 13 | 1 | 25 | 1 | 5 | 1 |
| Number of included predictors^b^ $\downarrow$ | | 17 | 18 | 16 | 18 | 18 | 10 | 17 | 1 |
| Brier score (95% Confidence Interval) $\downarrow$ | Training | 0.143 (0.142–0.144) | 0.141 (0.140–0.142) | 0.140 (0.139–0.141) | 0.139 (0.138–0.140 | 0.140 (0.139–0.141) | 0.140 (0.139–0.141) | 0.153 (0.152–0.154) | 2 |
|  | Temporal validation | 0.151 (0.148–0.154) | 0.150 (0.147–0.153) | 0.149 (0.146–0.152) | 0.149 (0.146–0.153) | 0.149 (0.146–0.152) | 0.149 (0.146–0.152) | 0.160 (0.158–0.163) | 1 |
|  | Geographical validation | 0.138 (0.135–0.141) | 0.136 (0.133–0.139) | 0.137 (0.134–0.140) | 0.138 (0.135–0.141) | 0.137 (0.134–0.140) | 0.137 (0.134–0.140) | 0.152 (0.150–0.154) | 2 |
| AUROC (95% Confidence Interval)^c^ $\uparrow$ | [Training](https://yanan-hu.github.io/fig/auroc_training) | 0.752 (0.750–0.755) | 0.768 (0.766–0.771) | 0.766 (0.764–0.769) | 0.772 (0.770–0.775 | 0.768 (0.766–0.771) | 0.766 (0.763–0.768) | 0.758 (0.756–0.761) | 3 |
|  | [Temporal validation](https://yanan-hu.github.io/fig/auroc_temporal) | 0.747 (0.737–0.755) | 0.757 (0.749–0.765) | 0.756 (0.748–0.765) | 0.756 (0.748–0.765) | 0.760 (0.751–0.769) | 0.757 (0.747–0.765) | 0.753 (0.744–0.762) | 2 |
|  | [Geographical validation](https://yanan-hu.github.io/fig/auroc_geographical) | 0.730 (0.720–0.739) | 0.751 (0.742–0.760) | 0.746 (0.737–0.755) | 0.745 (0.735–0.753) | 0.747 (0.738–0.757) | 0.747 (0.738–0.755) | 0.744 (0.736–0.753) | 2 |
| AUPRC (95% Confidence Interval)^c^ $\uparrow$ | [Training](https://yanan-hu.github.io/fig/auprc_training) | 0.392 (0.387–0.396) | 0.425 (0.420–0.430) | 0.423 (0.419–0.429) | 0.436 (0.431–0.441) | 0.430 (0.425–0.435) | 0.423 (0.418–0.429) | 0.405 (0.400–0.410) | 4 |
|  | [Temporal validation](https://yanan-hu.github.io/fig/auprc_temporal) | 0.407 (0.392–0.424) | 0.432 (0.416–0.452) | 0.430 (0.415–0.447) | 0.427 (0.413–0.445) | 0.434 (0.418–0.454) | 0.428 (0.412–0.447) | 0.427 (0.411–0.444) | 4 |
|  | [Geographical validation](https://yanan-hu.github.io/fig/auprc_geographical) | 0.328 (0.313–0.343) | 0.369 (0.352–0.386) | 0.349 (0.336–0.367) | 0.352 (0.337–0.369) | 0.358 (0.344–0.375) | 0.356 (0.341–0.373) | 0.355 (0.341–0.372) | 3 |
| Upper limit of well-calibrated predicted probability^c,d^ $\uparrow$ | [Training](https://yanan-hu.github.io/fig/calibration_curve_training) | 43% | 6% | 81% | 54% | 54% | 63% | 0% | 2 |
|  | [Temporal validation](https://yanan-hu.github.io/fig/calibration_curve_temporal) | 43% | 27% | 63% | 54% | 54% | 63% | 0% | 1 |
|  | [Geographical validation](https://yanan-hu.github.io/fig/calibration_curve_geographical) | 27% | 6% | 26% | 26% | 26% | 26% | 0% | 2 |
| Clinical utility^c^ (23% threshold probability) $\uparrow$ | [Training](https://yanan-hu.github.io/fig/decision_curve_training) | 7.50% | 7.59% | 7.74% | 7.91% | 7.77% | 7.75% | 7.09% | 3 |
|  | [Temporal validation](https://yanan-hu.github.io/fig/decision_curve_temporal) | 8.90% | 8.92% | 8.95% | 8.90% | 8.91% | 8.93% | 8.45% | 2 |
|  | [Geographical validation](https://yanan-hu.github.io/fig/decision_curve_geographical) | 4.94% | 5.01% | 5.23% | 4.98% | 5.14% | 5.22% | 4.64% | 2 |

Arrows indicate the favourable direction. Shaded cells represent the best value in their respective rows (i.e. across all algorithms). **^a^**Overall training time was based on our computer properties: Processor: Intel(R) Xeon(R) Gold 6242 CPU @ 2.80 GHz; Installed RAM: 8.00 GB. The included predictors and their importance rankings are available in Fig A. ^c^Click on the hyperlinked text to view interactive figures, where you can explore the x and y values of each data point by hovering over them and filter specific models by clicking on model names in the legend. A 23% threshold probability was chosen as an illustrative example (a reasonable preference as this is the prevalence of caesarean section following induction of labour across Australia in 2022[^8^](#_ENREF_8)). ^d^The difference between the observed and predicted probability of less than 3% is considered as well calibration.

Abbreviations: AUROC: Area Under the Receiver Operating characteristic Curve; AUPRC: Area Under the Precision-Recall Curve.

# Table G: Comparison of method and performance between the XGBoost model and previously validated models

| **Method or performance metrics** | | | **Hu 2025 Australia**  **(our XGBoost model)** | **Study ID (Author, Year, Country, Reference)** | | | | | |
| --- | --- | --- | --- | --- | --- | --- | --- | --- | --- |
|  |  |  |  | **Danilack 2020 USA**[^9^](#_ENREF_9) | **Rossi 2020 USA**[^10^](#_ENREF_10) | **Jochum 2019 France**[^11^](#_ENREF_11) | **Zhou 2022 China**[^12^](#_ENREF_12) | **Migliorelli 2019 Spain**[^13^](#_ENREF_13) | **Levine 2018 USA**[^14^](#_ENREF_14) |
| Training data | Sample size (cesarean section%) $\uparrow$ | | 180,700 (20.8%) | 17,370 (7.4%) | 4,177,644 (19.2%) | 1,024 (24.4%) | 2,950 (13.3%) | 338 (20.7%) | 491 (27.7%) |
|  | All inductions regardless of indication and cervical status | | Yes | No | Yes | No | No | No | No |
|  | Time range $\uparrow$ | | 2016–2019 | 2007–2012 | 2012–2016 | 2015 | 2014–2016 | 2014–2015 | 2013–2015 |
| Predictors | Number of included predictors $\downarrow$ | | 10 | 8 | 7 | 11 | 9 | 5 | 5 |
|  | All prior to induction | | Yes | Yes | Yes | No | No | No | No |
|  | All routinely collected | | Yes | Yes | Yes | No | No | No | No |
|  | No a priori selection | | Yes | Yes | No | No | No | Yes | No |
| AUROC (95% Confidence Interval) $\uparrow$ | | Training | 0.766 (0.763–0.768) | 0.818 (0.81–0.83) | 0.787 (0.786–0.788) | 0.76 (0.73–0.79) | 0.73 (0.70–0.75) | 0.826 (0.78–0.87) | 0.79 (0.74–0.83) |
|  |  | Temporal validation^a^ | 0.757 (0.747–0.765) | NA | 0.783 (0.764–0.802) | NA | 0.67 (0.64–0.70) | NA | NA |
|  |  | Geographical validation^a^ | 0.747 (0.738–0.755) | 0.824 (0.79–0.86)^c^ | NA | 0.81 (0.79–0.82)^d^ | NA | 0.735 (0.677–0.793^e^ | 0.73 (0.72–0.74)^d^ |
| Upper limit of well-calibrated predicted probability $\uparrow$ | | Training^b^ | 63% | 28% | 70% | 0% | 30% | NA | 50% |
| AUPRC (95% Confidence Interval) $\uparrow$ | | Training^b^ | 0.423 (0.418–0.429) | NA | NA | NA | NA | NA | NA |
| Brier score (95% Confidence Interval) $\downarrow$ | | Training^b^ | 0.140 (0.139–0.141) | NA | NA | NA | NA | NA | NA |
| Clinical utility | | | Yes | No | No | No | No | No | No |
| Performance across sociodemographic subgroups | | | Yes | No | No | No | No | No | No |
| Model accessible as a user-friendly tool | | | Yes | No | Yes | Yes | Yes | No | Yes |
| Comparison of various algorithms | | | Yes | No | No | No | No | No | No |

Arrows indicate the favorable direction. Shaded cells represent the best value in their respective rows (i.e. across all studies). ^a^Only validation studies using the same eligibility criteria of the development cohort were included. ^b^Only training performance was included due to all previous models did not report any of these performance metrics on training or validation cohorts. ^c^Validated using a hospital not included in the development cohort. ^d^Validated using a 2002-2008 USA birthing cohort. ^e^Validated using a 2019-2020 Spain birthing cohort by López-Jiménez 2022.[^15^](#_ENREF_15) Abbreviations: NA: Not Available; AUROC: Area Under the Receiver Operating characteristic Curve; AUPRC: Area Under the Precision-Recall Curve.

# References

1. Riley RD, Ensor J, Snell KIE, Harrell FE, Martin GP, Reitsma JB, et al. Calculating the sample size required for developing a clinical prediction model. BMJ. 2020;368:m441.

2. Collins GS, Ogundimu EO, Altman DG. Sample size considerations for the external validation of a multivariable prognostic model: a resampling study. Statistics in Medicine. 2016;35(2):214-26.

3. Yang C, Fridgeirsson EA, Kors JA, Reps JM, Rijnbeek PR. Impact of random oversampling and random undersampling on the performance of prediction models developed using observational health data. Journal of Big Data. 2024;11(1):7.

4. Hasanin T, Khoshgoftaar T, editors. The effects of random undersampling with simulated class imbalance for big data. 2018 IEEE international conference on information reuse and integration (IRI); 2018: IEEE.

5. Australian Bureau of Statistics. Standard Australian Classification of Countries (SACC). Canberra: ABS; 2016. Contract No.: 18 November.

6. Australian Bureau of Statistics. Remoteness Areas. Canberra: ABS; Jul2021-Jun2026. Contract No.: 18 November.

7. Australian Bureau of Statistics. Socio-Economic Indexes for Areas (SEIFA), Australia. ABS; 2021.

8. Australian Institute of Health Welfare. Australia's mothers and babies. Canberra: AIHW; 2024.

9. Danilack VA, Hutcheon JA, Triche EW, Dore DD, Muri JH, Phipps MG, et al. Development and Validation of a Risk Prediction Model for Cesarean Delivery after Labor Induction. Journal of Women's Health. 2020;29(5):656-69.

10. Rossi RM, Requarth E, Warshak CR, Dufendach KR, Hall ES, DeFranco EA. Risk Calculator to Predict Cesarean Delivery Among Women Undergoing Induction of Labor. Obstetrics & Gynecology. 2020;135(3):559-68.

11. Jochum F, Le Ray C, Blanc-Petitjean P, Langer B, Meyer N, Severac F, et al. Externally Validated Score to Predict Cesarean Delivery After Labor Induction With Cervi Ripening. Obstetrics & Gynecology. 2019;134(3):502-10.

12. Zhou H, Gu N, Yang Y, Wang Z, Hu Y, Dai Y. Nomogram predicting cesarean delivery undergoing induction of labor among high-risk nulliparous women at term: a retrospective study. BMC Pregnancy and Childbirth. 2022;22(1):55.

13. Migliorelli F, Baños N, Angeles MA, Rueda C, Salazar L, Gratacós E, et al. Clinical and sonographic model to predict cesarean delivery after induction of labor at term. Fetal Diagnosis and Therapy. 2019;46(2):88-96.

14. Levine LD, Downes KL, Parry S, Elovitz MA, Sammel MD, Srinivas SK. A validated calculator to estimate risk of cesarean after an induction of labor with an unfavorable cervix. American Journal of Obstetrics & Gynecology. 2018;218(2):254.e1-.e7.

15. López-Jiménez N, García-Sánchez F, Hernández-Pailos R, Rodrigo-Álvaro V, Pascual-Pedreño A, Moreno-Cid M, et al. Risk of caesarean delivery in labour induction: a systematic review and external validation of predictive models. BJOG. 2022;129(5):685-95.
