# Supplementary material for: Explainable machine learning model for predicting cesarean section following induction of labor: Development and external validation using real-world data
Source: PLOS Digit Health. 2025 Nov 20;4(11):e0001061. doi: 10.1371/journal.pdig.0001061 (PMC12633899; doi:10.1371/journal.pdig.0001061)
Supplement: Attachment — Submitted filename: Response to Reviewers.docx [file pdig.0001061.s004.docx]

**Response Letter**

**Title:** Explainable machine learning model for predicting cesarean section following induction of labor: Development and external validation using real-world data

We sincerely appreciate the time and effort the reviewers and editor have devoted to reviewing our study and providing insightful feedback. Below, we provide point-by-point responses to comments that required clarification or revision.

**Reviewer #1**

| **No.** | **Comment** | **Response** |
| --- | --- | --- |
| 1. | As far as the predictors and generalisability of their research goes, the main hinderance would be the usage of region to mask potential socioeconomic and associated health factors. Although these factors are important to consider, they as the authors themself state obscure factors such a gestation diabetes. | We have added the following limitations: “*Moreover, although women’s region of birth was included as a predictor, it may obscure more granular socioeconomic and health-related characteristics, such as education level, income, health literacy, cultural or religious practices, and access to care, which could further affect the generalizability of the model.”* |
| 2. | Prospective clinical trials will be required for validation and definition of cut-offs to the risk probabilities calculated. | We have added the following sentences to highlight the need for future research on threshold cut-offs: “*The results of the decision curve should not be used to set the range of threshold probability for the model. Instead, a pre-determined, clinically reasonable range of threshold probabilities should be used to evaluate which model has the highest net benefit across that range ^3^. In our case, the reasonable range of threshold probability for IOL against other labor management has not been determined, warranting future qualitative research involving clinicians and women*.” |

**Reviewer #2**

| **No.** | **Comment** | **Response** |
| --- | --- | --- |
| 3. | The missing data handling strategy of assigning arbitrary values (99 for continuous variables) rather than principled imputation methods is methodologically suboptimal despite low missingness (<3%). | We have added the following explanation to the Methods section to clarify why no data imputation was performed:  “*Due to the small proportion of missing data (< 3%) in some candidate predictors and complete data for the outcome (CS), no data imputation was conducted as recommended in the current literature.^1, 2^ Instead, missing values were grouped as “unknown” for categorical variables or assigned an unrealistic outlier value of 99 for continuous variables. Continuous variables were not categorized to avoid potential loss of information. This approach allowed the model to recognize missingness as potentially informative, while maintaining applicability in real-world settings where user inputs may also be incomplete.*” |
| 4. | The moderate class imbalance (20.8% cesarean rate) isn't explicitly addressed through appropriate techniques. | We originally explored the effect of both random oversampling and undersampling in our data, but given our sufficiently large sample size, neither approach improved model performance. This is consistent with the findings of previous methodological studies ^4, 5^, which also found minimal impact of these techniques in large observational health data and therefore did not recommend their use.  Due to word count limitations, details regarding the handling of class imbalance were not included in the main text. Instead, they are described in the Methods section of the Supplementary Material: “*No data balancing technique was applied as advised by the findings of prior comparison studies.^4, 5^*” |
| 5. | The manuscript would benefit from more detailed justification of why XGBoost was superior beyond performance metrics and better discussion of clinical implementation challenges. | We have added the following sentence to justify the selection of XGBoost: “*Although XGBoost did not rank first for each performance metric across all datasets, it consistently achieved robust AUROC, AUPRC, and other metrics, while using the fewest predictors (n = 10) and the shortest training time (1 hour). This balance of stable performance, parsimony, and computational efficiency enhances its practical value and real-world applicability.*”  Discussion of clinical implementation challenges has been added to the Discussion section as follows: “*Nonetheless, several challenges remain for the successful clinical implementation of machine learning–based decision support tools ^6^. Foremost among these are the limited explainability and transparency of complex algorithms, which constrain trust among both clinicians and women. In addition, the absence of standardized frameworks for evaluating algorithmic bias, fairness and explainability continues to impede widespread adoption. Integrating prediction models into existing electronic health record (EHR) systems presents further challenges, including compatibility with diverse data infrastructures and secure data exchange. Collectively, these barriers highlight the immaturity of current methodological and implementation science in this domain. Addressing them will require sustained interdisciplinary collaboration encompassing technical innovation, rigorous evaluation, user-centered design, ethical and regulatory governance, and policy development, before such models, including ours, can be deployed at scale in a reliable and responsible manner.*” |
| 6. | Additionally, while the comprehensive performance evaluation is excellent, the authors could strengthen their approach by considering interaction terms for key predictors, providing confidence intervals for SHAP values, and conducting sensitivity analyses comparing their missing data approach to proper imputation methods. | As noted in Table S2 (Description of used machine learning algorithms) in the Supplementary Material, the machine learning algorithms we applied intrinsically capture nonlinearities and interactions among predictors, and therefore do not rely on prior knowledge from researchers or experts to specify the model structure. Due to word count and figure/table limitations, this explanation is placed in the Supplementary Material.  Confidence intervals for the mean SHAP values of each predictor have been calculated using the 2.5th and 97.5th percentiles of the 1,000 bootstrapped samples and have been added to Figure 2A (also included below for easier reference).  The handling of missing data has been addressed in our response to Comment #1. |

**Figure 1:** Mean absolute SHAP contributions with 95% CI shown in black error bars

**Reviewer #3**

| **No.** | **Comment** | **Response** |
| --- | --- | --- |
| 1. | Given the CS rate (~20%) and resulting class imbalance, AUROC alone may be insufficient to reflect clinical utility. Please also report F1-score, sensitivity, specificity, and confusion matrices for both temporal and geographical validations. | We have added the following sentences to clarify why classification metrics were not presented at a single threshold cut-off: “*Recognizing that every woman may have a different risk tolerance, our models predict individualized probabilities of CS rather than making binary classifications (CS or not). Therefore, classification metrics at a single threshold are not presented. Instead, all combinations of these metrics across the full range of thresholds are made available through the interactive figures.*”  A screenshot of the sensitivity and specificity values at one randomly selected threshold is provided below as an illustrative example to demonstrate how these metrics can be retrieved from the interactive figures. |
| 2. | According to Table S6, XGBoost is not consistently the top performer in AUROC or AUPRC across datasets. Could you please clarify the decision criteria for selecting XGBoost as the final model? | We have revisited the following sentence to clarify how the final model was selected: “*Although XGBoost did not rank first for each performance metric across all datasets, it consistently achieved robust AUROC, AUPRC, and other metrics, while using the fewest predictors (n = 10) and the shortest training time (1 hour). This balance of stable performance, parsimony, and computational efficiency enhances its practical value and real-world applicability.*” |
| 3. | Were predictors chosen purely because they were available in the dataset, or based on prior literature and clinical relevance? Please cite supporting references for included variables where possible. | We have revisited the following sentence to clarify the selection of candidate predictors: “*Eighteen variables known before the start of IOL and available across the datasets were selected as candidate predictors. No a priori selection based on prior literature or clinical relevance was conducted, as predictor selection was data-driven.*” |
| 4. | The manuscript should explicitly describe how the outcome (CS) was ascertained from linked datasets (e.g., CPT codes or other computable phenotypes). | We have addressed this by adding the following clarification in the Methods section: “*Midwives and other birth attendants, using information obtained from mothers and from hospital or other records, complete notification forms for each birth, including labor type and mode of birth. CS was directly defined based on the mode of birth variable in the PDC data dictionary.*” Furthermore, the data collection manuals are available on the first author’s GitHub, with the link (<https://github.com/Yanan-Hu/CSAI/tree/main/Data%20dictionary>) provided in the Supplementary File and Data Sharing Statement. |
| 5. | There are two different front colors used in the text. | We thank the reviewer for their observation. The different font colour appears only in the in-text references and web links, which are automatically formatted as hyperlinks. These will be standardised by the journal’s editorial and production team during the publication process. |

**Figure 2:** An illustrative example showing how to view sensitivity (e.g. XGBoost = 72.6%) and specificity (e.g. XGBoost = 100% – 32.9% = 67.1%) from the interactive AUROC figure (source: <https://yanan-hu.github.io/fig/auroc_temporal>)

**References**

1.	Sisk R, Sperrin M, Peek N, van Smeden M, Martin GP. Imputation and missing indicators for handling missing data in the development and deployment of clinical prediction models: A simulation study. Stat Methods Med Res. 2023;32(8):1461-77.

2.	Gao S, Albu E, Stijnen P, Rademakers F, Cossey V, Debaveye Y, et al. Comparing methods for handling missing data in electronic health records for dynamic risk prediction of central-line associated bloodstream infection. arXiv preprint arXiv:250606707. 2025.

3.	Vickers AJ, van Calster B, Steyerberg EW. A simple, step-by-step guide to interpreting decision curve analysis. Diagnostic and Prognostic Research. 2019;3(1):18.

4.	Yang C, Fridgeirsson EA, Kors JA, Reps JM, Rijnbeek PR. Impact of random oversampling and random undersampling on the performance of prediction models developed using observational health data. Journal of Big Data. 2024;11(1):7.

5.	Hasanin T, Khoshgoftaar T, editors. The effects of random undersampling with simulated class imbalance for big data. 2018 IEEE international conference on information reuse and integration (IRI); 2018: IEEE.

6.	Antoniadi AM, Du Y, Guendouz Y, Wei L, Mazo C, Becker BA, et al. Current Challenges and Future Opportunities for XAI in Machine Learning-Based Clinical Decision Support Systems: A Systematic Review. Applied Sciences. 2021;11(11):5088.
